# Enhancing Clinical Diagnosis With Convolutional Neural Networks: Developing High-Accuracy Deep Learning Models for Differentiating Thoracic Pathologies

**DOI:** 10.7759/cureus.65444

**Published:** 2024-07-26

**Authors:** Kartik K Goswami, Nathaniel Tak, Arnav Wadhawan, Alec B Landau, Jashandeep Bajaj, Jaskarn Sahni, Zahid Iqbal, Sami Abedin

**Affiliations:** 1 College of Medicine, California Northstate University, Elk Grove, USA; 2 Medicine, Midwestern University Arizona College of Osteopathic Medicine, Glendale, USA; 3 School of Medicine, Wayne State University, Detroit, USA; 4 Internal Medicine, California Northstate University College of Medicine, Elk Grove, USA; 5 Radiology and Nuclear Medicine, Veterans Affairs (VA) Northern California Healthcare System, Mather, USA

**Keywords:** chest x-ray, general radiology, general internal medicine, x-ray analysis, thoracic radiology, deep learning artificial intelligence

## Abstract

Background

The use of computational technology in medicine has allowed for an increase in the accuracy of clinical diagnosis, reducing errors through additional layers of oversight. Artificial intelligence technologies present the potential to further augment and expedite the accuracy, quality, and efficiency at which diagnosis can be made when used as an adjunctive tool. Such techniques, if found to be accurate and reliable in their diagnostic acuity, can be implemented to foster better clinical decision-making, improving patient quality of care while reducing healthcare costs.

Methodology

This study implemented convolution neural networks to develop a deep learning model capable of differentiating normal chest X-rays from those indicating pneumonia, tuberculosis, cardiomegaly, and COVID-19. There were 3,063 normal chest X-rays, 3,098 pneumonia chest X-rays, 2,920 COVID-19 chest X-rays, 2,214 chest X-rays, and 554 tuberculosis chest X-rays from Kaggle that were used for training and validation. The model was trained to recognize patterns within the chest X-rays to efficiently recognize these diseases within patients to be treated on time.

Results

The results indicated a success rate of 98.34% incorrect detections, exemplifying a high degree of accuracy. There are limitations to this study. Training models require hundreds to thousands of samples, and due to potential variability in image scanning equipment and techniques from which the images are sourced, the model could have learned to interpret external noise and unintended details which can adversely impact accuracy.

Conclusions

Further studies that implement more universal database-sourced images with similar image scanning techniques, assess diverse but related medical conditions, and the utilization of repeat trials can help assess the reliability of the model. These results highlight the potential of machine learning algorithms for disease detection with chest X-rays.

## Introduction

In the realm of medical diagnostics, the integration of artificial intelligence (AI) technologies, specifically deep learning models such as convolutional neural networks (CNNs), has been instrumental in enhancing the accuracy and efficiency of pathology detection from medical images. This paper presents an advanced deep learning model developed for distinguishing normal chest X-rays from those indicative of various pathologies, including pneumonia, tuberculosis, cardiomegaly, and COVID-19. The model demonstrates notable accuracy, highlighting its potential to transform diagnostic processes.

The imperative for such innovation is supported by the expanding body of literature that underscores AI’s transformative role in medical imaging. Research conducted by Di et al. (2024) illustrates AI’s efficacy in classifying soft tissue tumors, positioning it as a valuable adjunctive diagnostic tool that augments the capabilities of pathologists [1]. Similarly, Gitto et al. (2024) explore the clinical applications of AI in musculoskeletal imaging, particularly its effectiveness in detecting fracture-related pathologies via sophisticated algorithms [2]. These studies are indicative of a broader trend toward the integration of AI technologies to facilitate more accurate and expedient medical diagnoses.

Additionally, Arabyarmohammadi et al. (2024) address the challenges associated with cardiac allograft rejection grading, advocating for AI-enabled digital pathology as a means to surmount the limitations inherent in conventional grading criteria [3]. This is substantiated by Alymai et al. (2024), who showcase AI’s ability to match and surpass traditional diagnostic methods when detecting fibrosis in Crohn’s disease through molecular imaging [4].

The study additionally considers the findings of Rogalla et al. (2024), who investigate the capabilities of AI visual aid tools in evaluating the resectability of pancreatic adenocarcinoma [5]. This underscores the pivotal role of AI in advancing medical diagnostics. Furthermore, Pokkuluri and Khang (2024) delve into the utilization of CNNs in precision medical imaging, highlighting the contribution of deep learning to personalized diagnostic techniques [6]. Lastly, the research by Xue et al. (2024) into AI-driven differential diagnosis of dementia using multimodal data demonstrates the extensive impact of AI across various medical scenarios, significantly enhancing diagnostic and therapeutic approaches [7].

The model developed in this project analyzes a comprehensive dataset of chest X-rays, each labeled for specific pathologies of pneumonia, tuberculosis, cardiomegaly, and COVID-19. Through an exhaustive training and validation regimen, the model achieved an accuracy rate of 98.34%, evidencing its exceptional proficiency in disease identification from medical images. This performance not only attests to the model’s robust design and the computational prowess afforded by an Nvidia RTX 3090 24GB graphics card but also underscores the expansive potential of AI in redefining medical diagnostic standards.

This project exemplifies the integration of AI in healthcare, presenting a model that not only achieves high diagnostic accuracy but also lays the groundwork for future innovations in medical imaging. As AI technology continues to evolve, its application in healthcare is anticipated to unveil new possibilities in patient care, heralding a future where diagnostics are increasingly informed by the precision and intelligence of artificial systems.

## Materials and methods

Objective

The project aimed to develop a deep learning model capable of differentiating between normal chest X-rays and those showing signs of pneumonia, tuberculosis, cardiomegaly, and COVID-19.

Dataset

The study utilized a comprehensive dataset comprising images labeled as normal, pneumonia, tuberculosis, cardiomegaly, and COVID-19. Due to patient privacy concerns and limitations on hospital databases, these images were collected from Kaggle databases [8-13]. The datasets used were chosen based on their organization of images, classifications of pathologic/nonpathological states, and labeling for better training of the model. Overall, there were 3,063 normal chest X-rays, 3,098 pneumonia chest X-rays, 2,920 COVID-19 chest X-rays, 2,214 chest X-rays, and 554 tuberculosis chest X-rays used for training and validation.

Data preprocessing

The images were preprocessed to normalize their sizes and intensities. Given the diverse nature of the diseases, specific augmentation techniques such as rotation, scaling, and flipping were applied to increase the dataset’s variability, aiming to improve the model’s generalization capability.

Model development environment

The model was developed using PyTorch, a leading deep learning framework, and executed on an Nvidia RTX 3090 24GB graphics card, providing significant computational power to handle the intensive training process. As this model is proprietary, the code has not been released for public access.

Training process

The model employed CNNs due to their proven effectiveness in image recognition tasks. The training process was divided into epochs, with each epoch involving a complete pass through the entire dataset. The training involved optimizing the model to reduce a loss function, measured as a combination of the training and validation losses, while monitoring accuracy and error rates to gauge performance.

Open science

This study did not receive any funding and the authors have no relevant financial disclosures. Due to the scrubbing of patient data from the datasets before they were uploaded to Kaggle, patient privacy was of less concern and did not require patient approval. Other than the public access datasets from Kaggle, there was no other patient or public involvement in this study. During this study, the protocol was not prepared.

## Results

**Figure 1:**
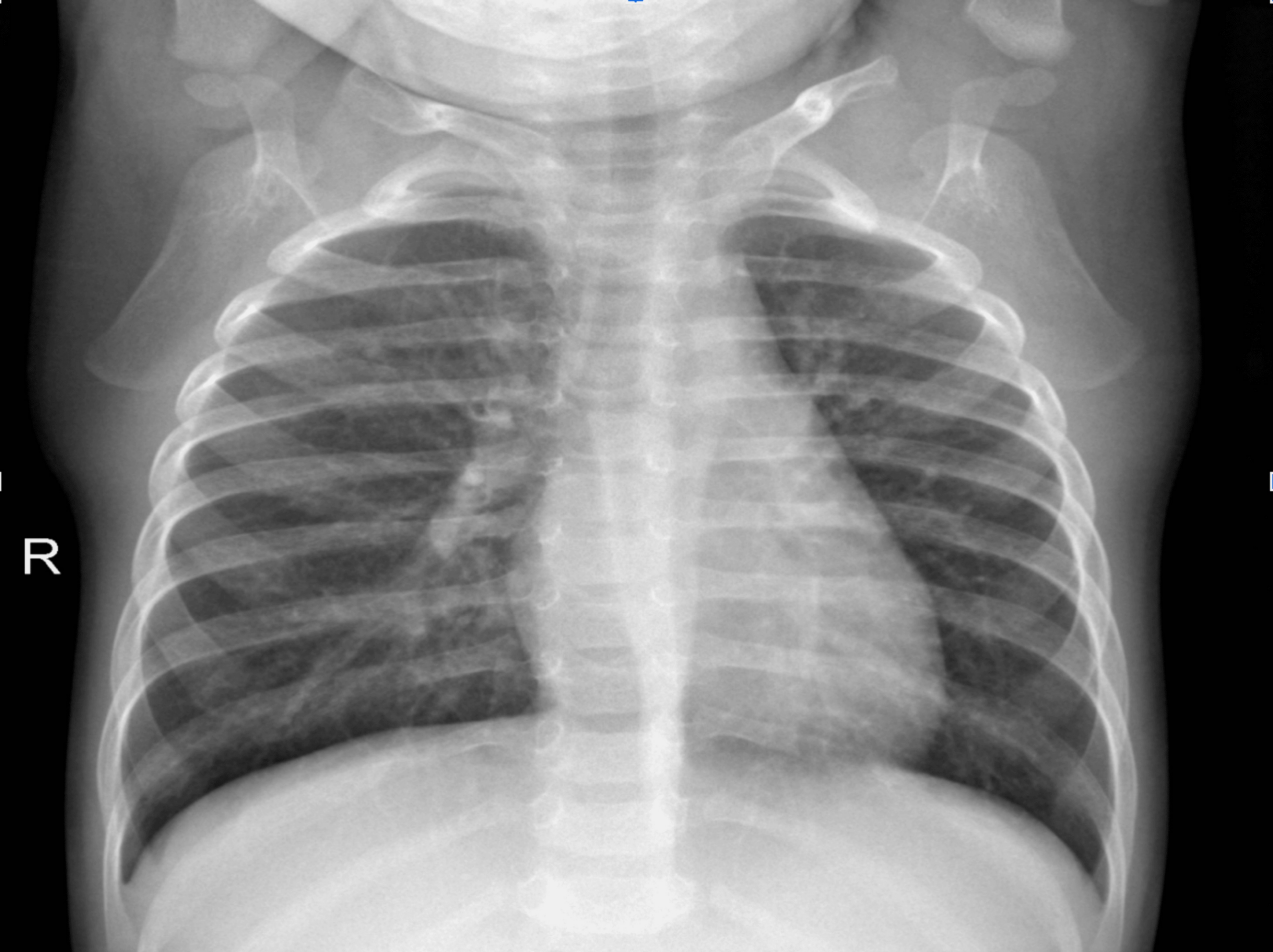
Normal chest X-ray. An example of the normal chest X-ray used to train our model. Chest X-ray images. Accessed on January 5, 2024, from https://www.kaggle.com/datasets/tolgadincer/labeled-chest-xray-images.

The model showcased outstanding performance in differentiating between normal chest X-rays and those showing signs of pneumonia, tuberculosis, cardiomegaly, and COVID-19. An example of this is shown in Figure 1 as a representation of what this model had inputted as a frame for learning pathologies in chest X-rays. The model achieved a remarkable accuracy of 98.34% which shows the model’s capability in accurately identifying the various conditions from chest X-rays.

**Figure 2:**
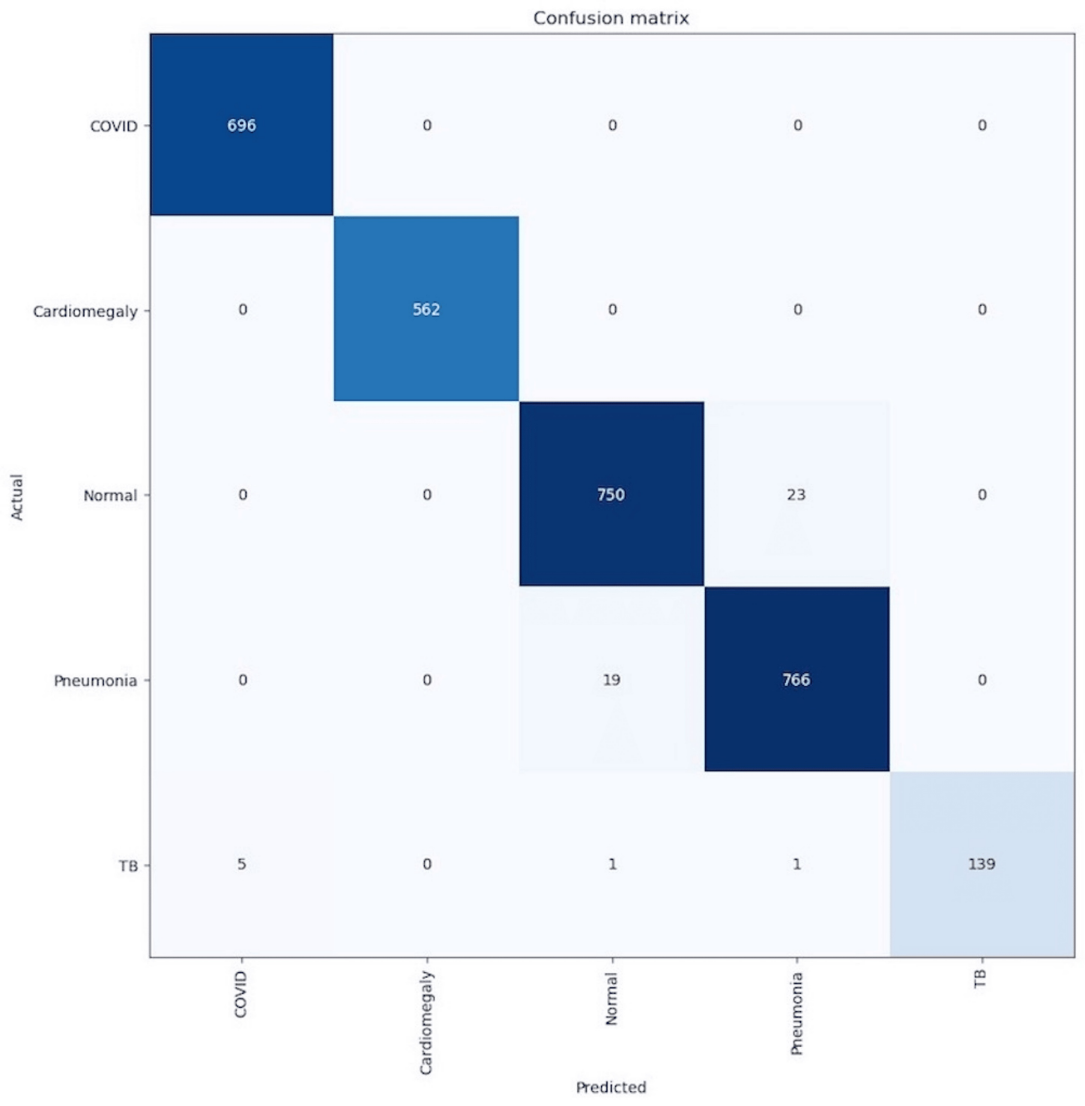
Confusion matrix. The figure shows our true positive, true negative, false positive, and false negative ratios calculated by our confusion matrix.

To further evaluate this model’s performance statistics of sensitivity, specificity, and overall accuracy were calculated using the provided values. The values were calculated in the confusion matrix, as shown in Figure 2, by finding the true positive, true negative, false positive, and false negative ratios.

**Figure 3:**
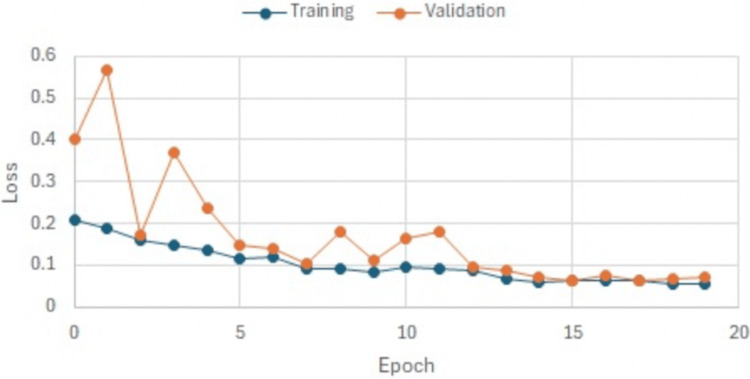
Run 1 predictive ability of the model. This graph shows our model’s accuracy and functionality during our first run.

**Figure 4:**
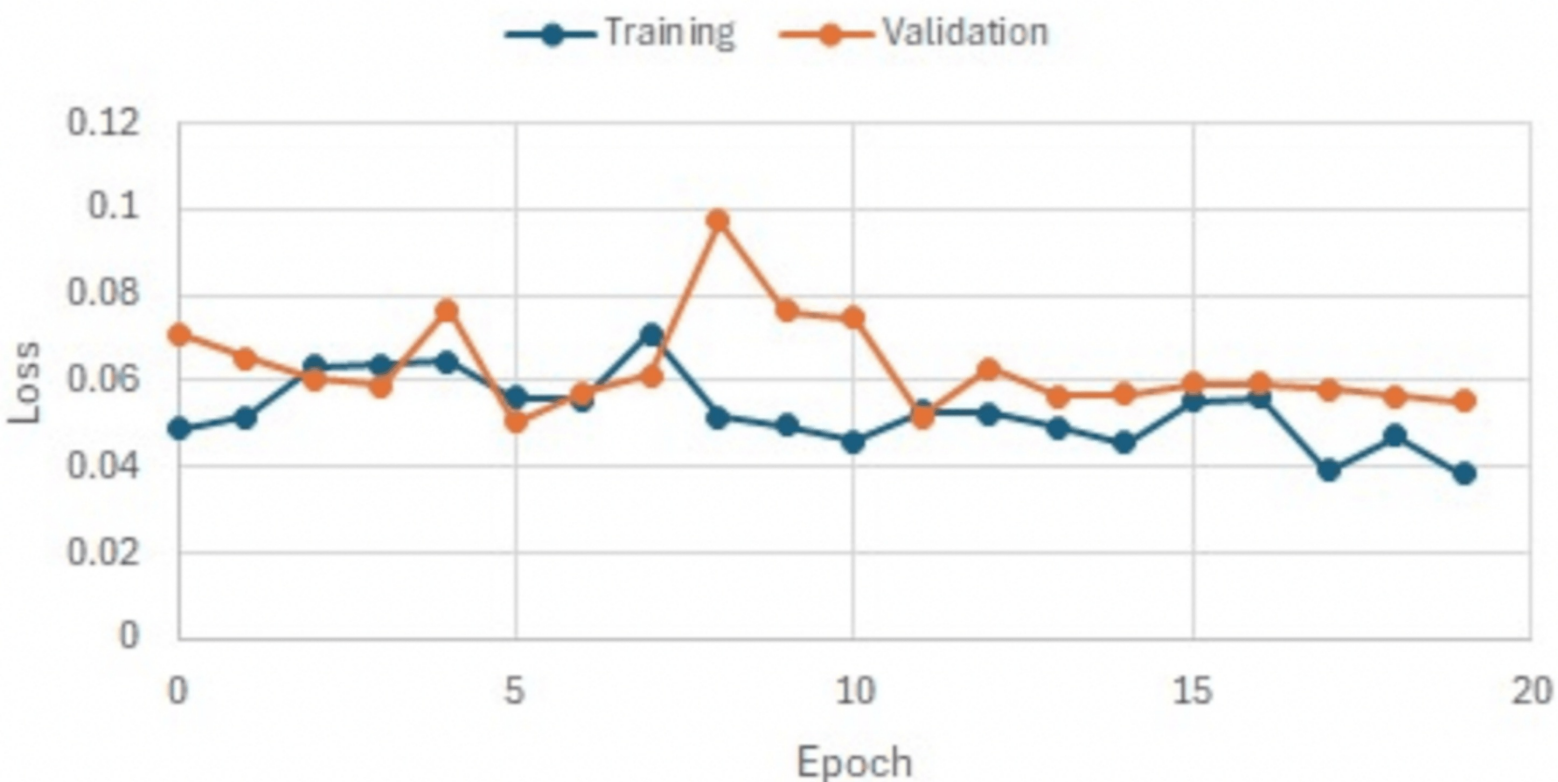
Run 2 predictive ability of the model. This graph shows the accuracy of our model improving on run 2 as the model learns from its previous run.

Throughout the training process, both training and validation loss metrics exhibited a decreasing trend across epochs, reflecting the model’s enhanced predictive ability with diminishing errors as training advanced. This is seen in Figure 3 and Figure 4 which show run 1 and run 2 of our training that improved the validation of our model. Furthermore, the error rate saw a significant reduction, further affirming the model’s effectiveness.

Leveraging an Nvidia RTX 3090 24GB graphics card, the model’s training was marked by high computational efficiency, allowing for quick completion of each epoch without sacrificing performance.

However, potential challenges were identified that could impact the model’s training and performance. The diversity of diseases analyzed meant the dataset could contain images of varying clarity, resolution, and quality. The variability introduced by different imaging equipment, techniques, and patient positioning may affect the model’s training and performance. Moreover, there is a concern regarding overfitting, a condition where the model, despite its reported high accuracy, might have learned the noise and details in the training data to a degree that it adversely affects its performance on new, unseen data. This highlights the importance of vigilance against overfitting to ensure the model’s robustness and reliability in real-world applications.

## Discussion

This study represents a significant step into the application of AI within the medical diagnostic field, utilizing CNNs to differentiate between normal chest X-rays and those indicative of pneumonia, tuberculosis, cardiomegaly, and COVID-19. Achieving an accuracy rate of 98.34% in detection, the model showcases not only its capability but also the potential for AI to redefine diagnostic processes in healthcare.

The precision of our model is underscored by its performance metrics, which reflect its ability to accurately identify the presence of specific pathologies in chest X-rays. The achieved accuracy rate of 98.34% is particularly compelling when considering the complexity and variability inherent in diagnosing these conditions. This is especially compelling when exploring other AI models such as CX-Ultranet presented by Kabiraj et al. (2024) which had an accuracy of 88% [14] and Hofmeister et al. (2024) which had an accuracy of 96% [15]. Such a high level of accuracy suggests that the model can serve as a reliable adjunct tool for healthcare professionals, potentially leading to quicker and more accurate diagnoses with little to no modifications of images.

However, the data also show challenges that require attention. Despite the high accuracy rate, the variability in the dataset, stemming from differences in image quality, imaging equipment, and techniques, poses a potential challenge to the model’s applicability across different clinical settings. This variability could introduce external noise, which, in turn, might affect the model’s learning process and diagnostic accuracy. For example, the training process revealed a gradual reduction in both training and validation loss, indicating an improvement in the model’s ability to generalize from the data it was trained on. Yet, this improvement necessitates a continuous evaluation of the model’s performance on external datasets to ensure its robustness and reliability.

Addressing the risk of overfitting is another critical consideration highlighted by the data. Overfitting occurs when a model learns the training data too well, including its noise and outliers, making it less effective at predicting new, unseen data. This was monitored through the validation loss metrics, which, while showing a decrease, required careful adjustment of the model’s parameters to ensure it did not memorize the training data but instead learned to generalize from it. This is underscored by Ahmad et al. (2023) who completed a systemic review of AI model papers and found some models experiencing drops in accuracy when analyzing external datasets due to overfitting on the training dataset [16].

Future directions for this research are informed by the data and the challenges encountered. The pursuit of standardized image collection and preprocessing methods appears vital to reducing variability and improving model performance across different settings. Moreover, expanding the dataset to include a broader spectrum of pathologies and imaging conditions could enhance the model’s diagnostic scope and applicability.

The data-driven insights from this study highlight the significant potential of AI, particularly CNNs, to augment the diagnostic process in healthcare. This statement is further supported by authors who have researched the use of AI in other fields such as Finzel (2024), who emphasizes the importance of human-centered AI explanations in improving the transparency and reliability of image classification tasks in digital pathology [17]. Further, Gouripur and Rashinkar (2024) elucidate the application of AI in detecting diabetic retinopathy, exemplifying its capability to advance diagnostic methodologies in pathology [18].

While the high accuracy rate achieved offers a glimpse into the future of medical diagnostics, the challenges identified underscore the need for ongoing research and development. With this study, there is a limitation of overfitting where the model may have learned to ignore the noise in the data, leading to potential adverse effects in unseen data. This highlights the importance of vigilance against overfitting to ensure the model’s robustness and reliability in real-world applications. By addressing these challenges and harnessing the power of AI, the goal of improving patient care through enhanced diagnostic accuracy and efficiency remains within reach.

## Conclusions

AI has been crucial to advancing medical diagnostics. The advanced deep learning model presented in this paper has demonstrated exceptional accuracy in distinguishing normal chest X-rays from those indicative of various pathologies, including pneumonia, tuberculosis, cardiomegaly, and COVID-19. The model’s impressive performance, achieving a remarkable accuracy rate of 98.34%, attests to its robust design and the computational capabilities harnessed through an Nvidia RTX 3090 24GB graphics card. AI’s increasing efficacy in studies on soft tissue tumors, musculoskeletal imaging, diabetic retinopathy, and cardiac allograft rejection grading showcases the versatile applications of AI in improving diagnostic accuracy and efficiency across various medical specialties. In addition to diagnostic accuracy, human-AI partnerships facilitate effective communication and clinical expertise. Despite these benefits, there is still much research to be done as potential challenges, including dataset variability, diverse imaging conditions, and the risk of overfitting could alter the reliability of the model in real-world applications.
